# Fine-Tuning Cold Stress Response Through Regulated Cellular Abundance and Mechanistic Actions of Transcription Factors

**DOI:** 10.3389/fpls.2022.850216

**Published:** 2022-03-29

**Authors:** Siti Nor Akmar Abdullah, Azzreena Mohamad Azzeme, Kobra Yousefi

**Affiliations:** ^1^Department of Agriculture Technology, Faculty of Agriculture, Universiti Putra Malaysia, Serdang, Malaysia; ^2^Laboratory of Agronomy and Sustainable Crop Protection, Institute of Plantation Studies, Universiti Putra Malaysia, Serdang, Malaysia; ^3^Department of Biochemistry, Faculty of Biotechnology and Biomolecular Sciences, Universiti Putra Malaysia, Serdang, Malaysia

**Keywords:** cold stress, transcription factors, CBF-dependent pathway, ICE1, chromatin modification, miRNA, REIL, ubiquitin proteosomic degradation

## Abstract

Inflictions caused by cold stress can result in disastrous effects on the productivity and survival of plants. Cold stress response in plants requires crosstalk between multiple signaling pathways including cold, heat, and reactive oxygen species (ROS) signaling networks. CBF, MYB, bHLH, and WRKY families are among the TFs that function as key players in the regulation of cold stress response at the molecular level. This review discusses some of the latest understanding on the regulation of expression and the mechanistic actions of plant TFs to address cold stress response. It was shown that the plant response consists of early and late responses as well as memory reprogramming for long-term protection against cold stress. The regulatory network can be differentiated into CBF-dependent and independent pathways involving different sets of TFs. Post-transcriptional regulation by miRNAs, control during ribosomal translation process, and post-translational regulation involving 26S proteosomic degradation are processes that affect the cellular abundance of key regulatory TFs, which is an important aspect of the regulation for cold acclimation. Therefore, fine-tuning of the regulation by TFs for adjusting to the cold stress condition involving the dynamic action of protein kinases, membrane ion channels, adapters, and modifiers is emphasized in this review.

## Introduction

Abiotic stresses such as low temperature, drought, and high salinity are complex quantitative traits where numerous stress-responsive genes including transcription factors (TFs) take part to ensure the survival of plants. The low temperature adversely affects plant growth and yield and significantly reduces crop efficiency. The TFs play a pivotal role in regulating eukaryotic gene expression, and several TFs families in plants are involved in regulating the expression of cold stress-responsive genes (Wang et al., 2020a). These TFs bind to the promoter regions of these genes to achieve a coordinated and effective response for cold acclimation. Depending on the types of TFs, different mechanisms are employed, thus affecting the selectivity and expression levels of the genes being regulated.

Plants respond differently for tolerance to low temperatures (0–15 degrees Celsius) and frost (below zero degrees Celsius). Adaptation to low temperatures is achieved by exposure to short-term cold, a process known as cold tolerance. Therefore, the cold tolerance can be defined as the ability of a plant to withstand low temperatures to prevent the stress from damaging its tissues (Ding et al., 2019). This process is associated with biochemical and physiological changes that will eventually lead to dramatic changes in gene expression, membrane lipid status, cell water uptake, membrane fluidity, and small molecules accumulation. Cold adaptation will increase the plant’s tolerance to physical and physicochemical changes when exposed to freezing temperatures. Plants in the tropical and subtropical regions however lack cold resistance mechanisms and they will suffer irreversible damage in the face of low temperatures (Knight and Knight, 2012). Plant resistance to low temperatures has a complex mechanism that involves different metabolic pathways in diverse cellular segments. Several genes have been identified to be involved in cold stress response, including genes that encode TFs, phosphatases, and kinases (Sun et al., 2018).

Plants show different reactions to cold stress where stress signaling pathways play a key role at the molecular and cellular levels. Signal detection, signal transmission, and stress response are three stages of signal transduction in plants when they receive stress stimuli. Possible mechanisms for coping with cold stress include binding of TFs to the cis-element in the promoter of cold stress-responsive genes, interactions of TFs with auxiliary proteins to facilitate their functions, regulation of reactive oxygen species (ROS), and signal transduction (Kumar et al., 2021). The stress response phase begins with phosphorylation and dephosphorylation events regulating the expression of these TF genes for protection at the cellular level. In addition, the expression of the TFs is regulated by microRNAs (miRNA) and their presence in the cells are subjected to translational and post-translational regulation. The different mechanisms will be described and discussed in the different sections of this review.

The TFs that are key players in plant response to cold stress including C-repeat Binding Factor (CBF)/Dehydration-responsive element-binding protein (DREB), Myeloblastosis (MYB), Basic helix–loop–helix (bHLH), NAC (NAM-ATAF1,2-CUC2), WRKY, and Basic leucine zipper (bZIP) gene families (Zhao et al., 2018) are shown in Table 1. The structures and activities of CBF/DREB, MYB, bHLH, and WRKY as well as their roles in regulating cold stress response are described in greater details as these TFs are mainly covered in this review.

**Table 1:** Transcription factor, cis-acting elements, and the corresponding responsive genes that are involved in plant abiotic stress.

No	TF families	Cis-acting element (Core Sequence)	Gene containing Cis-acting element	Species	Phenotypic changes in transgenic plant after induction of cold stress in comparison with wild type	References
1	CBF/DREB	DRE/CRT (A/GCCGAC)	*COR* genes	Sweet potato (*Ipomoea batatas* [L.] Lam)	Slight wilting, lower damaged leaves, little changed in the photosynthetic efficiency in transgenic sweet potato	Jin et al., 2017
2	NAC	CATGTG	Abiotic stress-responsive genes such as: *RD26*	*Arabidopsis thaliana*	Slightly chlorotic leaves with a larger leaf blade and shorter petiole than the wild-type plants	Nakashima et al., 2012
3	MYB	Type I Myb recognition sequences CNGTT(A/G) or type II G(G/T)T(A/T)GTT(A/G) and type IIG G(G/T)T(A/T)GGT(A/G)	Abiotic stress-responsive genes including *COR* genes	*Arabidopsis thaliana*	Induces darker green phenotypes in transgenic tobacco Enhances the contents of anthocyanins, phenolics, flavonoids, and soluble solids and, aroma volatiles in transgenic tomato	Agarwal et al., 2006; Yang et al., 2021; Ma et al., 2022
4	WRKY	W-box TTGACC/T	*RD29B*/*RD29A*/*COR6.6*/*DREB2A*	wild oat (*Avena fatua*)	Higher survival rates, increases in proline, lower electrolyte leakage in transgenic cucumber	Rushton et al., 2010; Zhang et al., 2016
5	bHLH	E-box (CANNTG) G-box (CACGTG)	*COR* and anthocyanin biosynthetic genes	Sweet potato (*Ipomoea batatas* [L.] Lam)	Slight damage under cold stress and returned to a normal state after recovery treatment in transgenic sweet potato	Jin et al., 2021; Shen et al., 2021
6	bZIP	ABRE (PyACGTG/TC)	*RD29A*, *RD29B*, *COR15A*, *COR47*	*Arabidopsis thaliana*	Lower ion leakages and higher survival in transgenic Arabidopsis	Wang et al., 2016
7	AP2 (ERF)	GCC-box (AGCCGCC)	Several abiotic stress-responsive genes	*Arabidopsis thaliana*	Greater survival rates of transgenic birch (*Betula platyphylla*) compared to wild type plant	Zhu et al., 2010; Lv et al., 2020

### C-Repeat Binding Factor/Dehydration-Responsive Element-Binding Protein

Plant stress TFs can control a set of genes by binding specifically to the cis-regulatory elements in the target gene promoters. The products of these genes act as stress response proteins, thereby increasing the plant’s tolerance to stress. CBF/DREB TFs are a large subset of the AP2/ERF family that play a key role in the expression of stress-responsive genes in the ABA-independent pathway. This family of TFs plays a vital role in regulating plant growth and response to external environmental stresses (Chen et al., 2013). At low temperatures, CBF activates the expression of cold-regulated (*COR*) genes which encode key enzymes for osmolyte biosynthesis and other cold stress-responsive genes by binding to the DRE/CRT (A/GCCGAC) cis-regulatory element (Vazquez-Hernandez et al., 2017). This enhances freezing tolerance through the accumulation of cryoprotective proteins and soluble sugars that repair cold-hardened membranes and stabilize cellular osmotic potential (Shi et al., 2018b).

The most well-known pathways for understanding and responding to cold stress in plants are the ICE-CBF-COR pathway involving 12% of all cold-responsive genes. This pathway is stimulated by low temperature, and with the induction of multiple intermediates step by step, eventually increases the expression of downstream genes regulated by CBF (Wang et al., 2020a). *ICE* (Inducer of CBF Expression) genes are at the forefront of the cold adaptation process, which induce the expression of *CBF* genes. When plants experience non-freezing low temperatures, the *CBF* genes are activated rapidly, and subsequently, the expression of downstream target *COR* genes referred to as regulons of CBF is induced. Thus far, there are four CBFs found in plants, and they are known as CBF1, CBF2, CBF3, and CBF4 representing DREB1B, DREB1C, DREB1A, and DREB1D (Alves et al., 2017; Liu et al., 2019; Xie et al., 2021a). The CBF1, CBF2, and CBF3 involved in cold response are sequentially located on chromosome four of Arabidopsis (Shi et al., 2018b). Mutations made on the *CBF* genes through the CRISPR/Cas9 system demonstrated the triple CBF mutants were more at risk of freezing (Zhao et al., 2016). Thus, indicates *CBF* genes are critical for cold adaptation. Therefore, further studies on CBF/DREB TFs will increase our understanding of applying beneficial strategies to improve plant tolerance to cold stress.

### Myeloblastosis

The MYB family is one of the most widespread plant TF families, containing above 100 members in Arabidopsis and rice involved in low temperature stress response. These TFs are divided into four different subtypes based on the structure of the DNA binding domain, which include 1R-MYB, R2R3-MYB, R1R2R3-MYB, and 4R-MYB (Su et al., 2014). This super family of proteins is involved in various processes such as cell cycle control, flower and seed development, primary and secondary metabolites regulation, hormonal signals, and biotic and abiotic stress responses (Li et al., 2015). Many studies have shown that the expression of *MYB* genes in response to cold and frost stress is dependent on the CBF/DREB pathway (Mehrotra et al., 2020). Transgenic plants that over-expressed *MYB* show enhanced expression of *CBF* genes with corresponding increase in tolerance to freezing stress before and after cold adaptation (Wang et al., 2014a).

MYB15 can be found in vegetative and reproductive organs of plants and plays a special role in regulating cold and salinity stress (Ding et al., 2009; Lindemose et al., 2013). MYB15 belongs to R2R3-MYB family of TFs in Arabidopsis that negatively regulates frost tolerance, which is brought about by its ability to repress the levels of expression of the *CBF* genes. MYB proteins are also involved in the interactions between cold, drought, and salinity stress responses (Li et al., 2015). Therefore, MYB proteins can be considered as an important regulator in plant responses to multiple abiotic stresses that can simultaneously control these abiotic stresses in higher plants. Current interest focuses on molecular mechanism associated with plant response to multiple stresses rather than focusing on a single type in isolation.

### Basic Helix–Loop–Helix

One of the important families of TFs in eukaryotes is bHLH, which has completely different functions in animals and plants. In plants, this TF family is active in regulating the expression of genes involved in hormonal and optical signals, stomata development, flowering, flavonoid biosynthesis, and response to biotic and abiotic stresses, including cold stress (Mehrotra et al., 2020). The protein sequence of this TF shows the presence of two functional domains, the HLH and basic domains, which together contain 60 conserved amino acids. The HLH domain with two alpha helices separated by a loop located at the C-terminal of the TF allows the interaction with other subunits to form homodimer and heterodimer. The basic domain with 15 amino acids at the N-terminal facilitates in binding to the G-box and E-box in gene promoters (Jin et al., 2021).

Studies in Arabidopsis and rice demonstrated the presence of 167 and 177 *bHLH* genes, respectively (Mao et al., 2017). Although limited information is available on this protein family in plants, studies have shown the involvement of bHLH in responding to abiotic stresses, including cold tolerance. *ICE1* and *ICE2* encode MYC-type bHLH TFs consisting of 496 amino acid residues. ICE1 binds to the MYC cis-element (CANNTG) at the promoter of *CBF1*/2/*3* to promote *CBF* genes transcription (Mehrotra et al., 2020; Sharma et al., 2020). NtbHLH123 transcription factor in tobacco is also a transcriptional activator that controls the expression of genes involved in ROS clearance, which led to the increase in cold tolerance (Zhao et al., 2018). In a study on sweet cherries, 66 *bHLH* genes were identified and most members of the PavbHLH family are associated with various processes in response to cold stress (Shen et al., 2021). The *IbbHLH* gene in sweet potato may also play a role in regulating cold stress, and *IbbHLH79* was identified as a potential candidate for plant molecular breeding to increase cold tolerance in sweet potatoes (Jin et al., 2021). These findings show the importance of bHLH in cold stress response. However, more studies are needed to reveal cold and ROS signaling pathways regulated by bHLH in plants.

### WRKY

The WRKY TF family in plants responds to a variety of abiotic stresses such as drought, salinity, and abnormal temperature. This family has undergone significant changes in higher plants during evolution, and the function of their genes has changed greatly from the original ancestral gene. Segmental and tandem duplications have also played a vital role in the development of this gene family. Previous reports indicate the essential role of WRKY TFs in managing low temperature stress. In tomatoes, for example, fragmentary duplication significantly contributed to the development of *WRKY* genes, and the expression of ten TFs from the WRKY family doubled during the period of cold stress (Chen et al., 2015). In a study on cucumber, over-expression of *CsWRKY6* increased cold tolerance as well as sensitivity to ABA and proline accumulation (Zhang et al., 2016). Recently, transcriptomic analysis in *Brassica napus* identified several genes of the WRKY family that play a vital role in cold resilience (Ke et al., 2020). Studies involving cold-resistant and cold-sensitive peanuts suggested that members from the NAC, MYB, and WRKY TF families were jointly involved in cold tolerance, indicating the importance of crosstalk between signaling pathways involving different TF families in response to cold stress (Jiang et al., 2020). The findings show WRKY enhances plant defense response to cold stress through ABA signaling pathway. It would be valuable to delineate the mechanism for cooperative involvement of the different TFs in cold stress response.

Many of the TFs involved in regulating cold stress response also play a role in other abiotic stress response pathways such as heat and drought. This is expected as most of the stress conditions lead to similar cellular disturbance especially excessive accumulation of ROS with damaging effects on macromolecules including carbohydrates, proteins, lipids, and DNA. Each TF recognizes a specific regulatory motif found on the promoters of different stress-responsive genes enabling simultaneous alteration in their expression (either up- or downregulation). The detection of adverse environmental condition is conveyed through plant signaling cascades to the TFs in order to achieve the desired cellular responses to overcome the damaging effects from the external environment as elaborated in the following sections.

## Transcriptional Reprogramming Involving Chromatin Modification and Heat Shock Factors

In response to cold stress, the production of ROS and NO mediates the alterations in chromatin structure and coordinates modification of histone and DNA methylation to activate the expression of stress-responsive genes (Kim, 2021; Tanpure et al., 2021). In eukaryotes, chromatin is organized as repeating subunits called nucleosome consisting of 147 bp DNA wrapped around a histone octamer containing four different histone proteins, H2A, H2B, H3, and H4 present in pairs. Loosening of the chromatin structure at a particular gene locus increases the accessibility of the transcriptional machinery and enhancing the transcriptional activity, which can be heritable and stably maintained between cell generations (Xie et al., 2021b). Histone acetyltransferase GENERAL CONTROL NON-REPRESSED PROTEIN5 (GCN5) catalyzes the acetylation of H3K9 and H3K14, which improves thermotolerance by activating heat stress-responsive genes, such as HEAT SHOCK TRANSCRIPTION FACTOR A3 (HSFA3), UV-HYPERSENSITIVE6 (UVH6), CHOLINE TRANSPORTER-LIKE 1 (CTL1), POLYGALACTURONASE INVOLVED IN EXPANSION3 (PGX3), and MYB54 (Hu et al., 2015; Zheng et al., 2019). The bivalent H3K4me3 and H3K27me3 marks found in active cold stress-responsive genes represent enhanced chromatin accessibility, which potentially allow access of proteins involved in transcriptional gene regulation (Zeng et al., 2019). The HIGH EXPRESSION OF OSMOTICALLY RESPONSIVE GENE15 (HOS15)-mediated chromatin modifications enable recruitment of CBF to *COR* gene promoters (Park et al., 2018).

The involvement of heat shock proteins (HSPs) in cold stress is still unclear compared to their action in heat stress. However, recent findings showed the role played by the HSPs (HSP90, HSP70, and smHSP) in controlling the intervention between heat and cold stresses in plants. Among HSPs, the HSP90 acts as a regulator of signal transfer to the nucleus, which is believed to be involved in reprogramming of transcriptional regulation of both stresses (Sohrabi et al., 2022). The regulation of HSPs expression is controlled by heat shock factors (HSFs). In plants, HSFs are categorized into three groups, the HSFA, HSFB, and HSFC. Among the three HSFs, the HSFA is involved in regulating HSPs expression and therefore activates multi-chaperone network. Under normal condition, the HSFA activity is negatively regulated by HSP90 while when responding to abiotic stress, the HSP90 is dissociated from the phospho-protein complex allowing interaction between HSFA and heat stress element (HSE) in the gene promoter region of many genes including those encoding antioxidants enzymes (Haq et al., 2019). The HSP90 was also reported to regulate cold stress response through resistance (R) proteins when pathogen attacks occurred under cold condition. This can be observed in Arabidopsis wherein HSP90 was found to be involved in the activation of RECOGNITION OF PERONOSPORA PARASITICA 4 (*RPP4*), one of the *R* genes in plants through SALICYLIC ACID GLUCOSYLTRANSFERASE 1-REQUIRED FOR Mla12 RESISTANCE 1-HEAT SHOCK PROTEIN 90 (SGT1-RAR1-HSP90) chaperone complex. The activity of SGT1-RAR1-HSP90 complex activated ENHANCED DISEASE SUSCEPTIBILITY 1 (EDSI)- and WRKY70-dependent cell death and defense response under cold condition (Bao et al., 2014). The findings show significant function of HSP90 in regulating cold signaling pathway even though under combined stresses of biotic and temperature stresses. In cold stress alone, increased HSP90 transcript was observed in *Brassica napus* (Krishna et al., 1995). However, in *Glycine max*, the downregulation of HSP90 was observed under cold condition compared to heat and salinity stress (Xu et al., 2013). This suggests the strong involvement of HSP90 in dehydration acclimation in *G. max* compared to cold stress. Recent findings in lentil further showed the involvement of other HSPs, the HSP70, HSP83, and HSP21 in mediating the cold response. Interestingly, these HSPs are also involved in heat stress response (Sohrabi et al., 2022). As cold and heat stress conditions do not occur concurrently in the plant, regulation of a specific HSP by HSF under cold and heat stress is potentially two independents events that assist plants to cope with abnormal temperature.

Stress memory programming at the transcriptional level is also important to enhance acclimation process toward cold stress in plants. This process involves the ability of primed plants to remember previous stress experience and therefore acquire them to enhance tolerance to similar or different stresses. It is a long-term effect that enables the plants to be protected from various stresses based on a previous encounter. In this process, the epigenetic modifications take place to mediate transcriptional memory, thus increasing plant adaptation to adverse temperatures (Xie et al., 2021b). As reported in Arabidopsis, the repetitive cold stress treatments reduced COR15A in resistance-improved plants. The reduction suggests the acclimation process, which could involve establishment of stress-induced H3K4me3 histone modification (Leuendorf et al., 2020). Increase in the expression of HISTONE DEACETYLASE 6 (*HDA6*) under cold stress in Arabidopsis had positively regulated freezing tolerance (Luo et al., 2017). Maize histone deacetylases (HDACs) may directly activate *ZmDREB1* gene expression and histone hyperacetylation under cold stress (Yu et al., 2018; Ding et al., 2019). Meanwhile, the regulation of expression of *COR* genes (*COR47* and *COR15A*) was reported to be regulated by HIGH EXPRESSION OF OSMOTICALLY RESPONSIVE GENE 15 (HOS15) and HISTONE DEACETYLASE 2C (HD2C) through direct binding to *COR* gene promoters (Park et al., 2018).

## Early and Late Cold Stress Response by Transcription Factors

**Figure 1:**
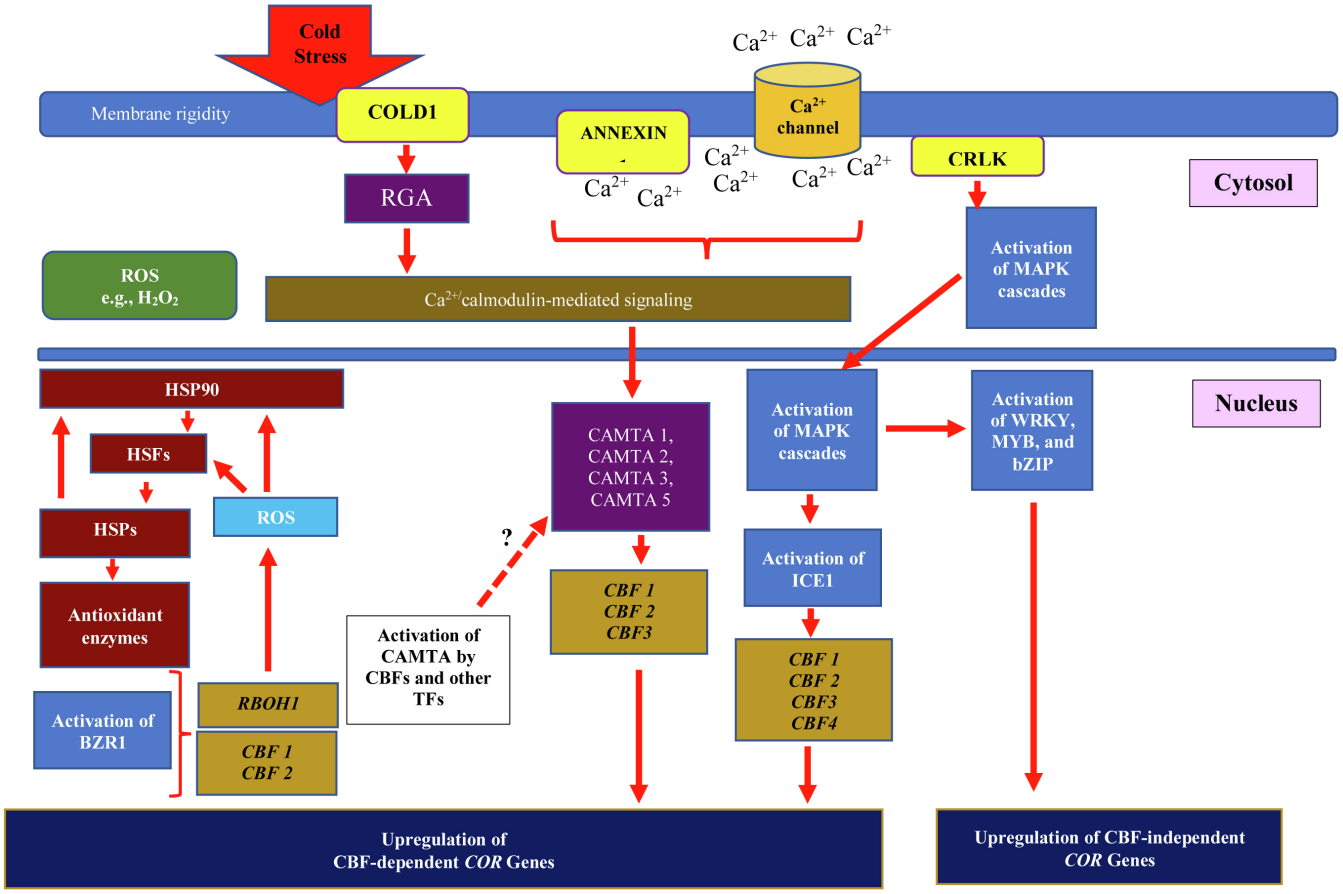
Integration of cold, heat, and ROS signaling in CBF-dependent and independent pathways for regulating plant cold stress response. Cold stress triggers changes in membrane fluidity and rigidity, and activates the expression of cold-regulated genes (*CORs*) through CBF-dependent and independent pathways. The chilling tolerance divergence 1 (COLD1) receives external cold stress signal and stimulates rice G-protein A subunit 1 (RGA1) activity. This further activates ANNEXIN1, the Ca^2+^ channels that transport Ca^2+^ into the cell. The COLD1/RGA activity and Ca^2+^ transmit cold stress signal to Ca^2+^/calmodulin-mediated signaling and Ca^2+^-dependent protein kinases (CDPKs) located in the cytosol. The CDPKs activities further convey cold stress signal through cytosolic and nuclear MAPKs signaling pathways, which leads to induction of ICE1 activity. ICE1 binds DRE/CRT motif in the promoter of *CBF 1*, *2*, *3*, and *4* and upregulates their expression. Integration of cold signal through Ca^2+^/calmodulin-mediated signaling activates interaction between CAMTAs and CM2 motif located in CBF promoter, which further upregulates *COR* genes expression. The presence of ABRE, SARE, G-box, W-box, AuXRE, and DRE motifs in *CAMTAs* promoters suggests the potential regulation of CAMTA by CBFs and other stress-responsive transcription factors (TFs). Accumulation of brassinosteroids promotes binding of BZR1 to E-box found in *CBF1* or *CBF2* genes and activates the expression of *COR* genes. Through CBF-independent pathway, the expression of CBF-independent *COR* genes is regulated through interaction of other TFs such as WRKY, MYB, and bZIP. The expression of CBF-independent *COR* genes could also be regulated through ROS signaling pathway, involving ROS produced by RESPIRATORY BURST OXIDASE HOMOLOG 1 (RBOH1). The ROS further regulate heat shock factors (HSFs) and heat shock protein 90 (HSP90) and hence develop multi-chaperone network that controls production of antioxidant enzymes. The multi-chaperone network is also involved in controlling HSP90 activity.

Modification of membrane proteins and activation of Ca^2+^ ion channels are plants primary responses when they sense cold stress from the environment. The chilling tolerance divergence 1 (COLD1) localized on plasma membrane and endoplasmic reticulum is the cold signal sensor that is responsible for receiving the cold signal from the environment (Figure 1). *COLD1* gene encodes a regulator of G-protein signaling, which causes the G-protein α subunit to activate the guanosine triphosphatase (GTPase) activity of rice G-protein A subunit 1 (RGA1; Guo et al., 2018). Ma et al., 2015 showed that over-expression of japonica rice *COLD1* enhanced chilling tolerance in rice compared to that of rice lines with downregulated expression of *COLD* 1. Once COLD1 receives the cold signal, elevation of cytosolic and nuclear Ca^2+^ occurs, and the plants activate calcium Ca^2+^ signaling pathway. Transportation of extracellular Ca^2+^ across plasma membrane through Ca^2+^ channels and/or Ca^2+^ pumps is triggered, increasing Ca^2+^ influx across plasma membrane (Yuan et al., 2018). The intracellular Ca^2+^ signal is then transduced through Ca^2+^ sensors, such as calmodulins (CaMs), CaM-like proteins (CMLs), Ca^2+^-dependent protein kinases (CPKs/CDPKs), and calcineurin B-like proteins (CBLs; Shi et al., 2018a). The Ca^2+^ sensors such as CaMs convey cold signal to TFs such as Calmodulin-Binding Transcription Activators (CAMTA). The promoter sequence of *CAMTA* genes possesses multiple cis-acting elements responsible for stress responses such as ABRE, SARE, G-box, W-box, AuXRE, DRE, and others (Noman et al., 2021), suggesting the potential interaction of cold-stressed responsive TFs with cis-acting elements of *CAMTA*. Upregulation of *CBF2* in Arabidopsis was reported due to interaction of Arabidopsis AtCAMTA3 to CM2 motif located in the *CBF2* promoter (Doherty et al., 2009). Kim et al., 2013 further reported the involvement of salicylic acid in inducing interaction between CAMTA1, CAMTA2, and CAMTA3 that further activated CBF1, CBF2, and CBF3 under cold stress. The decrement of salicylic acids was observed when the plants were exposed to warm temperature. Thus, CAMTAs play a critical role in the cold stress signaling cascade through binding to specific promoter motifs, which consequently influence the expression of *CBF* genes. However, based on the availability of multiple stress-responsive motifs located at *CAMTAs* promoters, multiples stress-responsive TFs can induce *CAMTAs* expression; therefore, accumulation of CAMTAs protein can enhance cold stress response through CBF-dependent signaling pathway. It further suggests the regulation of *CBFs* expression through CAMTAs other than that of ICE1 regulatory pathway.

Recently, Ca^2+^-permeable transporter ANNEXIN1 (AtANN1) localized in plasma membrane was reported to be involved in mediating the accumulation of cytosolic Ca^2+^ in response to accumulation of ROS. In this study, the research group found that the activated protein kinase, Open Stomatal 1 (OST1), phosphorylated AtANN1 at Ser289, and therefore enhances the Ca^2+^ transport activity and amplifies the Ca^2+^ signaling to activate the expression of *CBFs* and *CORs* in the nucleus (Liu et al., 2021). Moreover, the Ca^2+^ signal transduction through calcium/calmodulin-regulated receptor-like kinases (CRLK) activates the perception of downstream regulatory and signaling pathway through phosphorylation and activation of mitogen-activated protein kinases (MAPKs; Shi et al., 2018a). Two MAPKs are recognized in Arabidopsis, the MEKK1-MKK2-MPK4-CBFs and MKK5-MPK3/6-CBFs. Initiation of MEKK1 activity activates signal transmission to MPK4 through MKK1/2 by phosphorylation. Further, the activated MKK2 phosphorylates MPK4 and MPK6, and consequently activates physio-biochemical responses in cold-stressed plants (Chen et al., 2022). The MAPKs regulate the expression of cold-responsive genes including TFs, where their involvement in cold stress response can be distinguished as early (e.g., changes of membrane proteins and activation of ion channels) and late responses (i.e., cold acclimation; Wang et al., 2020d). Recently, studies by Shu et al. (2022) showed the involvement of ferulic acid in enhancing cold stress response in tomato. The exogenous application of ferulic acid decreased the severity of cold injury and upregulated the expression of *SlMAPK3*, *SlCBF1,* and *SlICE1*. Knockout mutant of *SlMAPK3* showed decrement of *CBF* expression in cold-stressed tomato suggesting the role of MAPK3 in *CBF* regulation.

The CBF coordinates cold signaling pathway through CBF-dependent regulatory pathway and CBF-independent signaling pathway (Park et al., 2018; Liu et al., 2019). In response to cold stress, the abscisic acid (ABA), jasmonic acid (JA), and indole acetic acid (IAA) are among the phytohormones involved in activating CBF-dependent signaling pathway (Wang et al., 2021; Zhou et al., 2021; Fu et al., 2022). The interaction between CBF and CRT/DRE motif regulates the downstream expression of an array of *COR* genes, which belong to *KIN* (cold induced), *RD* (responsive to desiccation), *LTI* (low temperature induced), and *ERD* (early dehydration inducible; Wani et al., 2021). The regulated products of *COR* genes include osmo-protectants biosynthetic enzymes, late embryogenesis abundant (LEA) proteins, TFs, protein kinases, proteins associated with lipid metabolism, proteins for hormone responses, cell wall modifiers, and chloroplast proteins (Liu et al., 2019). Discovery of an array of *COR* genes in Arabidopsis such as *COR6.6* (Gilmour et al., 1996), *COR15* (Lin and Thomashow, 1992), *COR47* (Gilmour et al., 1992), *COR78* (Horvath et al., 1993), and *COR413* (Breton et al., 2003) shows the important function of these genes in plant growth, development, and response to abiotic stresses.

In the early response to cold stress, the regulation of membrane fluidity of chloroplast is important for photosynthesis. The COR413 was reported to be involved in the regulation process. The COR413 TFs have been categorized into two groups, the Cor413-plasma membrane (COR413pm) and COR413-inner membrane (COR413im) proteins. The COR413im plays a role in stabilizing chloroplast membrane under cold stress. Meanwhile, over-expression of *Phlox subulata* PsCor413pm2 in Arabidopsis showed increased influx of Ca^2+^ in transgenic Arabidopsis root responding to cold shock. In addition, under cold stress, the increment of cytosolic Ca^2+^ in P*sCor413pm2* transgenic Arabidopsis increased the expression of five *AtCOR* (*AtCor6.6/AtKIN2*, *AtCor15A*, *AtCor15B*, *AtCor47*, and *AtCor78/AtRD29*) and two *AtCBF* (*AtCBF2* and *AtCBF3*; Zhou et al., 2018). Studies by Su et al. (2018) further revealed the involvement of COR413PM1 in regulating the expression of *COR* regulons in response to cold stress including the fatty acid biosynthesis 1 (*FAB1*), fructokinase 3 (*FRK3*), sucrose phosphate synthase A1 (*SPSA1*), and GLN phosphoribosyl pyrophosphate amidotransferase 2 (*ASE2*) in Arabidopsis leaves. Our previous studies showed that over-expression of oil palm *CBF1*, the *EgDREB1* enhanced tolerance to cold stress in lowland tomato. The EgDREB1 was suggested to control the expression of *COR* regulons such as *LePOD*, *LeAPX*, *LeCAT*, *LeGP*, *LeHSP70*, *LeLEA*, and *LeMET2.* Promoter analysis showed the presence of DRE/CRT motif in the promoter regions of these genes. EgDREB1 is also involved in drought stress signaling pathway as its expression was observed in transgenic tomato seedlings exposed to PEG treatment (Azzeme et al., 2017). These findings were similar with the observation carried out in oil palm, which we found that *EgDREB1* and *COR* regulons were also differentially upregulated in the drought-stressed oil palm (Azzeme et al., 2016). These findings, therefore, further suggest the involvement of EgDREB1 in both cold and drought signaling of oil palm. Similar findings were observed in Arabidopsis, where CBF/DREB1 that was known to be involved only in cold signaling was also induced under drought, ABA treatment, and salinity (Wang et al., 2014c). Lowland tomato over-expressing *EgDREB1* exhibited reduction in seed number, development of parthenocarpic fruits, alteration of leaves morphology, and increment of root biomass (Azzeme et al., 2020). Meanwhile, over-expression of *EgCBF3* increased the expression of antifreeze proteins, the *SlCHI3*, *SlPR1*, *SlPR-P2,* and *SlLAP2* in the lowland tomato. Delayed leaf senescence and flowering, increased chlorophyll content, and abnormal flowering were also recorded (Ebrahimi et al., 2016). These show involvements of CBF1 and CBF3 in plant growth and development, apart from their function as master regulators of abiotic stress.

### Differentiating CBF-Dependent and CBF-Independent Signaling Pathways

The expression of *COR* genes and their regulation either through CBF-dependent regulatory pathway or CBF-independent signaling pathway is still controversial. In Arabidopsis, the different alleles of the *CBF* gene were found to be controlling different regulatory mechanisms involving *COR* genes, and therefore resulting in different degrees of freezing tolerance. However, interestingly most of the *COR* genes found in this study were co-regulated by CBF-dependent and CBF-independent pathways (Park et al., 2018). It shows that multiple allelism could control different metabolic pathways in cold-stressed plants due to the presence or absence of CRT/DRE motif in the *COR* gene promoters. The absence of CRT/DRE motif indicates the activation of CBF-independent signaling pathway through other TF proteins like WRKY, MYB, and bZIP families to modulate plant responses to cold stress. For instance, Arabidopsis BRASSINAZOLE-RESISTANT 1 (BZR1) was reported to be involved in regulating the expression of *CBF1* and *CBF2* and other *COR* genes *via* CBF-independent pathway to modulate plant response to cold stress (Li et al., 2017). The regulation of BZR1 under cold stress is controlled by brassinosteroids accumulation. In tomato, BZR1 was found to interact with E-box/BRRE motifs located in *CBF1*, *CBF3*, and RESPIRATORY BURST OXIDASE HOMOLOG 1 (*RBOH1*) promoters and upregulated their expression. The activation of tomato ROS signaling pathway was achieved through the expression of *RBOH1*, in which the gene was found responsible in triggering H_2_O_2_. The H_2_O_2_ modulated the redox status in cold-stressed plants and increased BZR1 production and *CBF* genes transcription (Fang et al., 2021). Further, Ca^2+^ influx is also reported to be involved in the activation of *RBOH1*, hence enhancing the production of ROS (Kour et al., 2021). Apart from that, the expression of *CBF* could also be controlled by other TFs like ICE1 that activates *CBF* in response to cold stress. The mutation of ICE1 in Arabidopsis showed inhibition in expression of *CBF1*, *2,* and *3* and reduced resistance to cold stress (Chinnusamy et al., 2003). Conversely, the over-expression of *SlICE1* enhanced the expression of *SlCBF1* and increased the cold tolerance of tomato (Suzuki et al., 2012). The two-hour of cold exposure in transgenic potato over-expressing *SaMKK2* showed significant increase in the expression of *CBF1*, *2,* and *3* (Chen et al., 2022). In addition, a report by Jin et al., 2021 showed the involvement of bHLH (IbbHLH79) in activating sweet potato *IbCBF3*, through recognition of E-box (5′-CANNTG-3′) and G-box (5′-CACGTG-3′) located in the *IbCBF3* promoter, therefore enhancing tolerance to cold stress.

The crosstalk between CBF-dependent and -independent signaling pathways in plant response to cold stress enables a more coordinated regulation by the large network of TFs. Fine-tuning of transcriptional regulation by these TFs is achieved through regulating their cellular abundance and their activities as discussed further in the following sections.

## Regulated Expression of Transcription Factors by miRNA for Modulating Cold Stress Response

MicroRNAs (miRNAs) are recognized as a major gene family with an important role in gene regulation acting at the post-transcriptional level. First discovered in 1993, miRNAs are short endogenous single-stranded RNA of 20 to 24 nucleotides in length. These non-coding RNA molecules are derived from the typical stem loop precursors of 70 to 80 nucleotides long through the action of Dicer-like family of enzymes. Suppression of gene expression by miRNAs is achieved through two main mechanisms, which are mRNA cleavage and translation inhibition or combination of both (Megha et al., 2018). These mechanisms require binding of miRNAs to the target sites in mRNAs through perfect or near perfect base-pairing and there can be multiple mRNA targets for each miRNA. In plants, the target sites are mostly in the open-reading frame (ORFs) but some are found in the 5′-untranslated regions (UTRs) and 3’-UTRs of the mRNAs (German et al., 2008). Many of the miRNAs target genes encode TFs involved in transcriptional regulation of different plant developmental processes and stress responses. This constitutes an effective fine-tuning measure as the effects are extended to the various downstream genes regulated by these TFs.

Regulation by miRNA is critical for achieving the complex temporal and spatial expression profiles of each regulatory TF and the downstream genes being regulated by the TF (Samad et al., 2017; Wang et al., 2019a). The predicted target genes of miRNA under cold stress in winter turnip rape mainly encode for TFs, such as MYB, GAMYB, Teosinte branched 1/Cycloidea/Proliferating cell factor (TCP), bHLH, and SQUAMOSA promoter binding proteins (SBP; Zeng et al., 2018). The differential regulation of the TFs by the miRNA leads to cellular outcome essential for plant to cope with the cold stress condition. For example, integrated small RNA and transcriptome analysis of *Populus simonii* × *P. nigra* subjected to cold stress identified differential expression of miR319, miR159, miR167, miR172, miR395, miR393, miR390, and novel_63 and TFs including *MYB*, *SBP*, *bZIP*, Auxin Response Factors (*ARF*), and LONESOME HIGHWAY (*LHW*, atypical bHLH). These miRNAs and TFs direct or indirectly regulate the expression of Leucine-Rich Repeats (*LRR*) receptor kinase, DnaJ-related photosystem II, *ARF,* and *SPLs* associated with chilling injury (Zhou et al., 2019). In the construction of plant cold-responsive Gene Regulatory Network (GRN), the number of affected targets increased with the inclusion of TFs, which behave as central nodes for relaying information from miRNAs downstream to the TF regulons (Tiwari et al., 2020). Gene ontology enrichment analyses showed over-representation of distinct functional modules such as cold stress, transcription and translation, transport and pentatricopeptide repeat (PPR), cell wall and lignin synthesis, and signaling and protein degradation in the GRN.

### Key miRNAs Involved in Regulating Transcription Factors for Cold Acclimation

MiRNAs are conserved among plant species and their expression varies depending on environmental biotic and abiotic conditions. The regulation of expression of targeted TFs by miRNAs is dependent on the miRNAs’ tissue-specific expression and the differential effects of the cleavage on the target transcripts (Megha et al., 2018). miR166 and miR319 showed altered expression in the roots of winter turnip rape under cold stress compared to normal condition suggesting their pivotal roles in cold stress response. The strong downregulated expression of miR319 (miR319e-1, miR319a, and miR319–2) and miR166 (miR166e-3p) in the roots of cold tolerant compared to cold-sensitive cultivars has important implication as root tolerance is critical during winter for plants’ survival (Zeng et al., 2018). It was found that the miR319 from winter turnip rape targets TCP4-like which controls secondary cell wall formation (Sun et al., 2017) while the miRNA166 regulates HD-ZIP III with a key role in cell wall and cellulose synthesis (Zhang et al., 2018). Significantly higher expression of both TFs demonstrated in the cold-tolerant cultivar compared to cold-sensitive cultivar showed that improvement in cell wall thickness and strength is important for cold acclimation.

Significantly higher expression of *COR*s including *DREB1A/B/C*, *DREB2A*, and *TPP1/2* was observed in rice plants over-expressing Osa-miR319b, which targets GAMYB and TCP TFs. The increase in cold tolerance was evident based on the increase in proline content and survival rate of transgenic plants. It was suggested that miR319 negatively regulated *OsPCF6* and *OsTCP21* expression in rice, which partially increased the ROS scavenging capacity, therefore facilitating rice response under cold stress (Wang et al., 2014b). Over-expression of sha-miR319d from wild tomato silenced GAMYB-like1 and conferred chilling tolerance in cultivated tomato (Shi et al., 2019). Differential expression of key regulatory genes involved in chilling (*CBF1* and *MYB83*), heat stress response (*HSFA1a, HSFA1b,* and *HSP90*), and ROS signaling [zinc-finger proteins (*ZAT12* and *ZAT10*) and scavenging, superoxide dismutase (*SOD*), and catalase (*CAT*)] was observed in the transgenic compared to WT plants. This suggests that sha-miR319d regulates temperature stress in tomato *via* interaction of cold stress, heat stress, and ROS signaling pathways.

In rice, OsmiR156 plays an important role in enhancing tolerance to cold stress. Enhancement in cell viability and growth rate under cold stress was observed in Arabidopsis, pine, and rice over-expressing rice miRNA156 that targets *OsSPL3* involved in upregulating the expression of *OsWRKY71* (Zhou and Tang, 2019). Suppression of *OsWRKY71* enhanced the expression of *OsMYB2* and *OsMYB3R-2*. OsMYB2 is involved in regulating multiple stress responses including cold stress (Yang et al., 2012). OsMYB3R-2 transgenic rice showed enhanced tolerance to chilling stress due to the increase in expression of stress-responsive genes and the alteration in cell cycle (Ma et al., 2009).

However, a contrasting result was reported with OsmiR156k, probably due to the production of a different mature miRNA. OsmiR156k is a precursor miRNA which is differentially processed from the other precursor miRNAs (OsmiR156a-j; Moreal et al., 2016) and this may contribute to its differential accumulation and distinct function in plant tissues. Phenotypic analysis found that transgenic rice lines over-expressing OsmiR156k showed seedling growth inhibition at the very early seedling stage under cold stress. Lower survival rates, as well as reduction in chlorophyll and proline contents resulting from the ectopic expression of OsmiR156k, were observed. Downregulated expression of the *CORs* and *SPL3*, *SPL14*, and *SPL17*, the targets of OsmiR156k, was also detected (Cui et al., 2015).

Clearly, the comparison of miRNA expression between cold-tolerant and cold-sensitive cultivars and the functional studies in transgenic plants provided useful insights on regulatory mechanisms for cold acclimation involving miRNA156, miRNA166, and miRNA319 that play key roles in cold stress response in plants. The findings are summarized in Table 2.

**Table 2:** Functional studies involving miRNA156, miR166, and miR319. The target transcription factors of the miRNAs, effects on the expression of downstream or cold stress-responsive genes regulated by the transcription factors are provided. The observed phenotypic effects of the miRNA regulation under cold stress are also included.

miRNA	Target transcription factors	Observed effects on downstream or other cold stress-responsive genes	Phenotypic observation	References
miR156 (OsmiR156)	*SPL3*	Suppression of *OsWRKY71* leading to enhanced expression of *OsMYB2* and *OsMYB3R-2*.	Enhancement in cell viability, growth rate and reduction in ion leakage under cold stress in Arabidopsis, pine, and rice over-expressing rice OsmiRNA156	Zhou and Tang, 2019
miR156k (Osmi156k)	*SPL3*, *SPL14* and *SPL17*	Suppression of proline synthase and ROS scavenger genes	Seedlings’ growth inhibition at the very early stage, lower survival rates, lower ROS scavengers, reduction in chlorophyll and proline contents in rice over-expressing OsmiR156k	Cui et al., 2015
miR166 and miR319	*HD-ZIP III and TCP4-like*	N/A	Downregulated expression of miR166 and miR319 in roots under cold stress led to increase expression of HD-ZIP III and TCP4-like that potentially improve cell wall thickness and strength in the cold-tolerant cultivar.	Zeng et al., 2018
miR319 (Osa-miR319b)	*TCP21*, *PCF5* and *PCF6*	Higher expression of *CBF/DREB1*	Increase in proline, plant survival rate and ROS scavenging capacity under cold stress of transgenic rice over-expressing Osa-miR319b	Wang et al., 2014b
miRNA319 (sha-miR319d)	*GAMYB-like1*	Promote expression of genes involved in chilling (*CBF1* and *MYB83*), heat stress response (*HSFA1a, HSFA1b* and *HSP90*), and ROS signaling (*ZAT12* and *ZAT10*) and scavenging (*SOD CAT*)	Lower relative electrolyte leakage and malondialdehyde concentration, reduced O^2−^ generation and H_2_O_2_ concentration and higher chlorophyll contents and Fv/Fm values in cultivated tomato cultivar over-expressing sha-miR319d from wild tomato	Shi et al., 2019

## Controlled of Transcription Factor Levels Through Differential Protein Synthesis and Degradation During Cold Stress

### Ribosome Biogenesis Factors in Accelerating *de novo* Protein Synthesis in Cold Acclimation

An effective cold stress response is dependent on controlled cellular levels of specific TFs along the cold signaling pathway through mechanisms that regulate their synthesis and degradation. This influences the cascade of events through the cold signaling pathway enabling fine-tuning of the cold stress responses. Protein translation which occurs at the ribosome is an important step in the production of a functional cellular proteins in living organisms. Ribosome assembly is a complex process which requires coordination of the activities of three RNA polymerases and more than 200 transiently associated ribosome biogenesis factors (RBFs; Sáez-Vásquez and Delseny, 2019). Arabidopsis zinc-finger proteins, REILs are cytosolic ribosomal 60S-biogenesis factors with potential role in accelerating ribosome *de novo* synthesis (Wang et al., 2017; Beine-Golovchuk et al., 2018). Studies utilizing Arabidopsis *REIL* mutants provided valuable insights on the factors influencing ribosome biogenesis and function under cold stress. Findings of the study by Cheong et al., 2021 suggested that biosynthesis of specialized ribosomes is required for cold acclimation.

It was demonstrated that *STCH4/REIL2* helps in maintaining rRNA processing and promotes translation of CBF for regulating cold stress response (Yu et al., 2020). Over-expression of *STCH4/REIL2* in Arabidopsi*s* can confer chilling and freezing tolerance possibly through modified association of STCH4 with multiple ribosomal proteins. The reduction in rRNA processing ability exhibited by *stch4* Arabidopsis mutants was further worsened by cold stress treatment. The key observations of the mutants include reduction in the level of CBF and delayed induction of the CBF regulons. Clearly, REILs play a role in enhancing cold stress tolerance through altering ribosomal composition and functions, which promotes translation of proteins essential for growth and survival of plants under the adverse effects of cold stress (Yu et al., 2020; Cheong et al., 2021). Studies involving Arabidopsis double mutants of *REIL* complemented by systems analyses of transcriptome and metabolome of the ribosomal complexes (Wang et al., 2017; Beine-Golovchuk et al., 2018) suggest that REILs likely serve as kinetic modulators of ribosome biogenesis or recycling that may assist in overcoming the initial cold-induced inhibition of translation.

### Post-translational Regulation for Selective Degradation of Transcription Factors for Fine-Tuning Cold Stress Response

The 26S proteasome degrades TFs that have been covalently linked to a polyubiquitin chain to enable selective reduction in the abundance of a specific TF. Polyubiquitin chain is covalently attached to the target proteins through three sequential steps involving three enzymes; Ub-activating enzyme (E1), Ub-conjugating enzyme (E2), and Ub ligase (E3). The degradation of selective TFs is controlled strictly by numerous E3 Ub ligases responsible for substrate recognition. CULLIN-REALLY INTERESTING NEW GENE (RING) E3 ligases (CRLs) are the largest group of E3 ligase in plants. The multisubunit CRL regulates numerous biological processes through targeted ubiquitylation of signaling proteins. The four components of CRL core are the cullin scaffold protein, a RING finger protein that binds to an E2 ubiquitin conjugating enzyme, a receptor that recognizes the target protein, and adaptor proteins linking the receptor to the cullin. Different targets are recruited through binding of a large pool of distinct substrate-receptor modules to the N-termini of cullins (Lydeard et al., 2013; Wang et al., 2020c).

The ability to bind to the respective TFs that serve as substrates is crucial for the E3 Ub ligases activity and it is affected by other post-translational modifications including phosphorylation and SUMOylation (Buetow and Huang, 2016; Ding et al., 2019). Regulating the expression of CBF through controlling 26S proteasomic degradation of upstream TFs that act as regulators in the CBF-dependent signaling appeared as one of the key mechanisms for fine-tuning cold stress responses (Shi et al., 2018b). The increase or decrease in cellular abundance of a TF which acts as a positive or negative regulator influences the outcome of the cold signaling pathway.

In Arabidopsis, it was found that cold activation of ICE1 TF, the positive regulator of CBF3/DRB1A, is negatively affected by ubiquitination by RING E3 ubiquitin ligase HIGH EXPRESSION OF OSMOTICALLY RESPONSIVE GENE 1 (HOS1) which leads to its degradation through the 26S proteosomic degradation pathway (Ishitani et al., 1998). Similarly, cold tolerance is negatively regulated in banana by MaSINA1 which is an E3 ubiquitin ligase that targets MaICE1 (Fan et al., 2017). OPEN STOMATA 1 (OST1) is an important protein kinase involved in the regulation of plant response to cold stress (Ding et al., 2015). Under cold stress, it interacts with and phosphorylates ICE1, preventing it from interacting with HOS1. Stabilization of ICE1 promotes the transcriptional activity of CBF for *COR* induction. While under prolonged cold stress, activation of BRASSINOSTEROID-INSENSITIVE 2 (BIN2) kinase leads to phosphorylation of ICE1 which promotes ICE1 interaction with HOS1 and its subsequent degradation. This suppresses CBF activity and expression of *COR* genes (Ye et al., 2019). Thus, both kinases which act at different phases following cold stress encounter serve as partial regulators of *CBF* indirectly through ICE1. It is also believed that fine-tuning of *CBF* expression by BIN2 is important for balancing cold tolerance and plant growth (Ye et al., 2019). Thus, the regulation of the cellular abundance and activities of the CBF is important for plant adaptation and survival.

U-box E3 ligases (PUB25 and PUB26) from Arabidopsis target the upstream negative regulator of CBF/DREB1, MYB15. Phosphorylation of PUB25 and PUB26 by OST1 enhances their activities and the reduction in MYB15 level leads to increase expression of *CBF* genes which enhances the plant cold stress response (Wang et al., 2019b). Anthocyanin is essential for the cold acclimation response. Increase in anthocyanin improves antioxidant capability resulting in an increase in plant tolerance to low temperature (Naing et al., 2018). MYB TFs interact with bHLH TFs in regulating plant growth and development (Wang et al., 2020b). Apple MdbHLH33 activates the expression of *MdCBF2* and for regulating cold tolerance and anthocyanin accumulation. An et al. (2020) reported on MdMYB308L interaction with MdbHLH33 for enhancing binding to *MdCBF2* and *MdDFR* promoters. However, MYB30-INTERACTING E3 LIGASE 1 (MdMIEL1), an apple RING E3 ubiquitin ligase, was found to be interacting with MdMYB308L. The interaction which promotes degradation of MdMYB308L suppresses cold-tolerant response and anthocyanin accumulation in apple.

### Other Mechanisms Modulating 26S Proteosomic Degradation of Cold Stress-Responsive Transcription Factors

SUMOylation can modulate the activity of TFs through regulating the localization and abundance of TFs as well as by influencing their interaction with chromatin. SUMOylation protects conjugated protein from degradation by blocking the lysine residues that can be ubiquitinated (Roy and Sadanandom, 2021). Thus, SUMOylation of ICE1 by SAP AND MIZ1 DOMAIN-CONTAINING LIGASE 1 (SIZ1; Jmii and Cappadocia, 2021) protects ICE1 from degradation through the 26S proteosomic degradation pathway. Expression of *CBF/DREB1* and the downstream *COR* genes was increased while the expression of *MYB15* was suppressed due to SIZ1 conjugation of ICE1, enhancing the tolerance to low temperature (Miura et al., 2007). The fine-tuning of the CBF/DREB1 signaling pathway through post-translational SUMOylation and ubiquitination events involving the positive regulator ICE1-like was also observed in apple. The apple *MdCIbHLH1* which encodes an ICE1-like protein showed induced expression under cold stress. It can be modified through ubiquitination and SUMOylation pathways and binds to MdCBF2 for enhancing cold tolerance *via* the CBF signaling pathway. The ability of MdCIbHLH1 in maintaining its functionality in distantly related species, *Nicotiana tabacum* (tobacco; Feng et al., 2012), may suggest a universal mechanism for cold stress response in plants.

**Figure 2:**
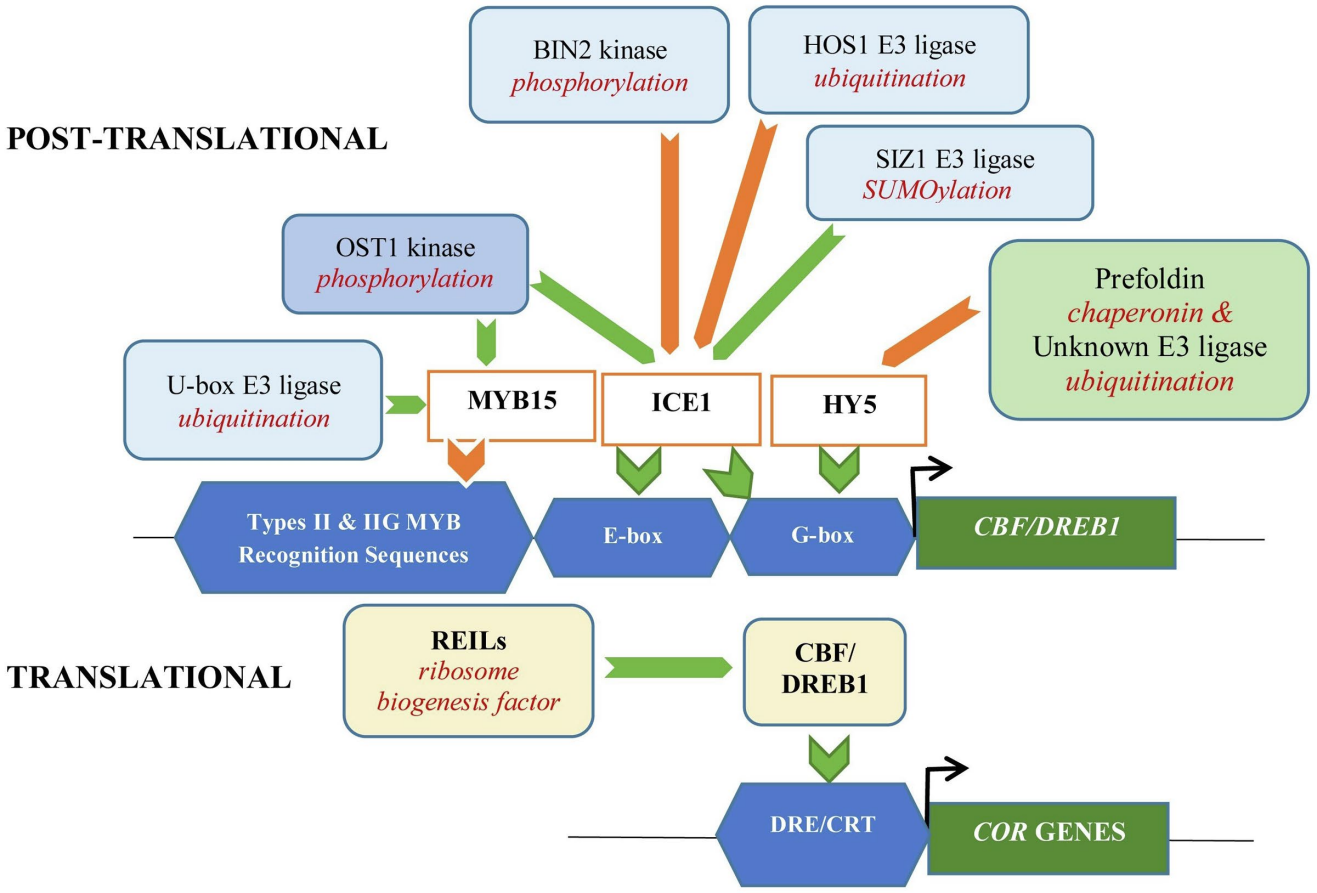
Translational and post-translational modifications of transcription factors affecting CBF-dependent signaling for cold acclimation in *Arabidopsis thaliana*. Translational regulation by ribosome biogenesis factor, REILs increase rRNA processing and CBF levels, positively affecting cold acclimation. Post-translational regulation through ubiquitination for proteosomic degradation of ICE1 (positive regulator of CBF) is suppressed by SUMOylation involving SIZ1 E3 ligase. Phosphorylation of ICE1 by OST1 disrupts its interaction with HOS1 E3 ligase preventing degradation of ICE1 and enhancing cold tolerance. While under prolonged cold stress, phosphorylation of ICE1 by BIN2 kinase facilitates interaction of ICE1 and HOS1 and subsequent degradation of ICE1. Phosphorylation of U-box E3 ligase (PUB25 and PUB26) by OST1 increases ubiquitination and degradation of the negative regulator MYB15 in Arabidopsis, enhancing the expression of CBF and its regulons, *COR* GENES. In contrast, promotion of ubiquitination of the positive regulator, HY5 through interaction with prefoldin which acts as a molecular chaperon, suppresses *CBF* expression. Green and orange arrows represent reactions/interactions that provide positive and negative effects, respectively, on the signaling pathway and expression of *COR* GENES.

Long Hypocotyl 5 (HY5), a bZIP TF which acts as a positive regulator of cold acclimation, has been shown to activate about 10% of all cold-induced transcripts including anthocyanin biosynthetic genes, facilitating in the complete development of cold acclimation in Arabidopsis. Recent report showed that HY5 can directly regulate the expression of *CBF 1*, *2,* and *3,* or indirectly *via* MYB15 as it was shown to bind to *MYB15* promoter region (Zhang et al., 2020). It has been reported that HY5 is subjected to post-translational control by COP1 E3 ubiquitin ligase (Catalá et al., 2011). Chaperonins are molecular chaperons involved in protein assembly, folding, trafficking, and degradation, with critical role in cellular development (Hartl et al., 2011). Prefoldin is a hexameric molecular chaperone belonging to group II chaperonins (Cao, 2016). Perea-Resa et al. (2017)demonstrated negative regulation of cold stress response in Arabidopsis by prefoldin which destabilizes HY5. Interaction of prefoldin, which accumulates in the nucleus with HY5, triggers ubiquitination and HY5 subsequent degradation through 26S proteosomic degradation pathway. This attenuated anthocyanin biosynthesis, which helped ensure accurate development of cold acclimation. Interestingly, the study showed that degradation of HY5 occurred in COP1-independent manner (Perea-Resa et al., 2017). Based on the findings, it is anticipated that prefoldin contributes indirectly through participation in cellular proteostasis to regulate the stability of TFs or complexes involved in different stages of gene expression (Blanco-Touriñán et al., 2021). Figure 2 provides a schematic diagram on translational and post-translational modifications affecting CBF-dependent signaling for cold acclimation that has been discussed.

## Summary and Future Perspective

The crosstalk between multiple signaling pathways in plant response to cold stress enables access to a large network of TFs as transcriptional regulators. Fine-tuning through regulation of the cellular abundance and activities of the TFs in the GRN is important for plant adaptation and survival. The expression of TFs is affected by intracellular accumulation of secondary messengers like Ca^2+^, ROS, NO, and phytohormones. Activities of the secondary messengers that regulate rearrangement of chromatin and coordination of histone modification and DNA methylation allow plants to express specific TFs during cold stress. The spatial and temporal regulation by highly conserved miRNAs such as miRNA156, miRNA166, and miRNA319 that modulates the expression of target TFs is important for fine-tuning the cold stress response across plant species. In Arabidopsis, the production of CBF, the key TF for regulating the expression of COR genes is enhanced during cold stress through interaction of REILs, the ribosome biogenesis factors with multiple ribosomal proteins that influence ribosome composition and RNA processing. Other TFs like ICE, HY5, and MYB15 that serve as negative or positive regulators of ICE-CBF-COR signaling cascades are selectively degraded through the proteosomic degradation pathway. This provides the essential cellular metabolic balance for a proper cold stress response. It also helps overcome retardation in plant growth associated with CBF expression. Post-translational modifications of these TFs including through phosphorylation and SUMOylation events or attachment of molecular chaperons, which block or promote binding of the respective E3 ubiquitin ligases, regulate their degradation, with major consequence on the expression of COR genes.

The CBF-independent BZR1 signaling cascades involving other TF families such as WRKY regulate the induced production of specific sets of COR genes independent of the DRE/CRT motif, the recognition site for CBF. BZR1 and HSFA play a central role in linking the cold stress signaling with ROS signaling and heat stress signaling pathways, respectively, through a complex mechanism involving other TFs, HSPs, and ROS scavenger genes. While CAMTAs which possess multiple cis-elements recognized by a variety of stress-responsive TFs serve central position in the cold stress signaling cascades. CAMTAs integrate inputs from these TFs and convey them downstream for a coordinated expression of the different COR genes. Together, the CBF- and CBF-independent pathways allow plants to adjust to the varying severity of cold stress. The plants could also enhance their defense response by memory reprogramming at the transcriptional level based on previous cold stress encounters.

In general, plants have developed fine-tuning mechanisms for understanding temperature fluctuations to increase their chances of survival. Comprehensive understanding of the fine-tuning of spatial and temporal expression of genes and their translational and post-translational regulation associated with cold stress is critical to produce stress-resistant plants through genetic engineering or genome-assisted breeding strategies for increasing crop yield. It is also of critical importance for extending geographical distribution of crops and for survival under extreme seasonal conditions due to the effects of climate change. Studies on REILs, which are still confined to the model plant, *Arabidopsis thaliana*, can be expanded to economically important crops through powerful multi-omics strategy and functional studies utilizing gene editing technology. Functional analysis of the different TFs could benefit from CRISPR/dCas9 activation and suppression systems to unravel the complex GRN in endogenous system which could provide valuable additional information compared to utilizing model plant systems.

## Author Contributions

SA produced the overall concept of the review, led the manuscript preparation, carried out the overall editing of the content, and she finalized the write-up. SA, AA, and KY contributed in writing and producing the tables and figures. All authors contributed to the article and approved the submitted version.

## Funding

Funding for the research was provided by the Ministry of Higher Education Malaysia under the Long-Term Research Grant Scheme (LRGS) LRGS/1/2020/UPM/01/2/2.

## Conflict of Interest

The authors declare that the research was conducted in the absence of any commercial or financial relationships that could be construed as a potential conflict of interest.

## Publisher’s Note

All claims expressed in this article are solely those of the authors and do not necessarily represent those of their affiliated organizations, or those of the publisher, the editors and the reviewers. Any product that may be evaluated in this article, or claim that may be made by its manufacturer, is not guaranteed or endorsed by the publisher.
